# Synergistic Interfacial Design of Cation Exchange Membranes via Sequential Electro-Assembly for High-Efficiency Lithium Separation

**DOI:** 10.3390/membranes16030087

**Published:** 2026-02-28

**Authors:** Zhibo Zhang, Geting Xu, Yangbo Qiu, Junbin Liao, Tong Mu, Wanji Zhou, Yunfang Gao, Jianquan Weng, Jiangnan Shen

**Affiliations:** 1 College of Chemical Engineering, Zhejiang University of Technology, Hangzhou 310014, China; 211123010172@zjut.edu.cn (Z.Z.); 201806022717@zjut.edu.cn (G.X.); jbliao@zjut.edu.cn (J.L.); 211123010202@zjut.edu.cn (T.M.); 2 State Key Laboratory of Advanced Separation Membrane Materials, National Key Laboratory of Green Chemical Synthesis and Transformation Technology, Zhejiang University of Technology, Hangzhou 310014, China; 3 Department of Civil Engineering, The University of Hong Kong, Hong Kong SAR 999077, China; ybqiu@hku.hk; 4 Salt Lake Chemical Engineering Research Complex, School of Chemical Engineering, Qinghai University, Xining 810016, China; wanjizhou@qhu.edu.cn

**Keywords:** cation exchange membrane, in-situ modification, lithium–magnesium separation, electrodialysis, monovalent selectivity

## Abstract

The industrial application of modified ion-exchange membranes is limited by complex, discontinuous ex-situ processes. This study introduces an in-situ electro-assembly strategy that enables the direct fabrication of a selective layer within an electrodialysis stack without disassembly. By utilizing a programmed current reversal to orchestrate the sequential deposition of polyethyleneimine (PEI), glutaraldehyde cross-linking, and polystyrene sulfonate (PSS) adsorption, we achieve meticulous interfacial engineering on a commercial cation exchange membrane. Comprehensive characterization confirms the successful construction of a hydrophilic, charge-tuned multilayer, which enhances ion transport kinetics and raises the limiting current density. This method culminates in a membrane with an exceptional Li^+^/Mg^2+^ selectivity of 107.9 and robust stability, retaining a significant selectivity of 47 over 10 cycles in real salt lake brine. This synergistic integration of operational simplicity, interfacial precision, and superior performance establishes a transformative and scalable platform for manufacturing high-performance membranes for selective ion separation from complex brine sources.

## 1. Introduction

The rapid expansion of lithium-ion batteries for electric vehicles, consumer electronics, and grid storage has led to a sharp rise in global lithium demand [1,2,3,4]. Salt lake brines, particularly those in the Qinghai-Tibet Plateau of China, represent an important lithium resource [5,6,7]. However, their extremely high Mg^2+^/Li^+^ ratios [8,9,10,11] and the similar physicochemical properties of Mg^2+^ and Li^+^ make selective lithium extraction highly challenging [12]. Electrodialysis (ED) offers notable advantages over conventional extraction methods due to its ability to separate monovalent and multivalent ions using permselective ion-exchange membranes (IEMs) [13,14,15,16,17,18,19,20,21,22]. Despite its high efficiency and low environmental footprint [23,24], the performance of ED remains constrained by the limited Li^+^/Mg^2+^ selectivity and insufficient long-term stability of commercial cation exchange membranes (CEMs) under harsh brine conditions, such as high salinity, elevated Mg^2+^/Li^+^ ratios, complex multi-cation compositions, and prolonged exposure to strong electric fields [25,26,27].

Numerous surface-modification strategies, including interfacial polymerization [28,29,30], chemical grafting [31,32], cross-linking, and electrostatic layer-by-layer (LbL) assembly [28,33,34,35], have been explored to improve CEM performance. These approaches enhance ion selectivity by tuning surface charge and introducing steric and electrostatic sieving effects [36,37,38,39]. Polyamine-based coatings and sulfonated polyelectrolytes have demonstrated enhanced monovalent-ion transport and suppressed multivalent-ion permeability [40,41], while multilayer poly(sodium 4-styrenesulfonate (PSS)/PAH membranes have achieved K^+^/Mg^2+^ selectivities above 300 due to hydrated-size exclusion and solvation-energy differences [42,43].

However, a major barrier prevents these advances from being translated into industrial ED systems. Most reported strategies rely on multi-step, ex-situ modification procedures requiring repeated membrane removal, immersion, rinsing, and re-assembly [44,45]. Such discontinuous procedures are time-consuming, labor-intensive, and fundamentally incompatible with continuous large-scale membrane fabrication, resulting in limited reproducibility and poor scalability.

To address this challenge, we introduce an in-situ electro-assembly strategy that enables continuous membrane modification directly inside an ED stack without any disassembly. By programming a sequence of current reversals, polyethyleneimine (PEI) electrodeposition, glutaraldehyde (GA) cross-linking, and PSS adsorption are sequentially orchestrated to construct a robust PEI-GA-PSS-PEI-GA interfacial layer. Unlike conventional multistep ex-situ routes, this method allows continuous preparation under realistic electro-driven conditions, significantly simplifying the fabrication process while enhancing membrane stability and industrial applicability [46,47,48,49,50]. In addition, most previous modification studies have evaluated membranes only in simplified salt solutions rather than real brines, where high salinity and complex ionic compositions often lead to performance deterioration during long-term operation. This further underscores the necessity of an in-situ electro-assisted strategy capable of both membrane fabrication and performance assessment under realistic brine conditions.

In this work, we detail the in-situ modification process, comprehensively characterize the physicochemical properties of the modified membranes, and systematically evaluate their separation performance and long-term stability under realistic brine environments. Beyond demonstrating a high-performance membrane, this study establishes a scalable and fundamentally new platform for on-demand engineering of functional interfacial layers, thereby bridging the gap between material design and industrial ED operation for selective lithium extraction [50,51,52].

## 2. Experimental

### 2.1. Materials

Commercial CEMs and anion exchange membranes (AEM) were purchased from [Zhejiang Baichen Low Carbon Technology Co., Ltd., Shaoxing, China] and used as the base membranes without further pretreatment, unless otherwise specified. PEI (molecular weight ≈25 kDa, purity ≥99%), GA (25 wt% aqueous solution), and PSS (average molecular weight ≈70 kDa, purity ≥98%) were obtained from [Beijing Bailingwei Science and Technology Co., Ltd., Beijing, China]. All chemicals were used as received without additional purification.

The feed solution for ED was prepared using either raw salt lake brine from the Dongtai Salt Lake in Ge’ermu, China.

### 2.2. Membrane Surface Modification

The surface modification of CEM was conducted in-situ within a four-compartment ED device made in our own laboratory. The base CEMs were first immersed in deionized (DI) water for 12 h to ensure complete hydration while maintaining the membranes in the H^+^ form. The pristine CEM (unmodified) was denoted as CEM-Basic.

Unless otherwise stated, the effective membrane surface area exposed during the modification process was 189 cm^2^, and the volume of each modification solution was 1000 mL.

#### 2.2.1. PEI Electrodeposition

The CEM-basic was positioned between the dilute compartment (DC) and concentrate compartment (CC). PEI solution (0.15 g·L^−1^, prepared in 25.7 g·L^−1^ NaCl solution) was introduced into the DC and 25.7 g·L^−1^ NaCl solution in CC. Electrodeposition was carried out in the reverse current mode at a current density of 5 mA·cm^−2^ for 4 h at room temperature. The resultant CEM was denoted as CEM-PEI. Then it was followed by a 5-min rinse with DI water to eliminate excess reactants and ensure controlled layer growth. During the electro-assisted modification process, the PEI-containing solution was introduced into DC without circulation, and electro-driven transport dominated the interfacial assembly process.

#### 2.2.2. GA Cross-Linking

The membrane CEM-PEI was positioned between the DC and CC. GA solution (0.10 g·L^−1^ in 25.7 g·L^−1^ NaCl solution) was supplied to the DC, with 25.7 g·L^−1^ NaCl solution in CC. Then the reverse-current modification was applied at 0.5 mA·cm^−2^ for 2 h at 40 °C to facilitate cross-linking between GA and PEI. The resultant CEM was denoted as PG. Then it was followed by a 5-min rinse with DI water to eliminate excess reactants and ensure controlled layer growth.

Although GA is electrically neutral, the electric current was maintained during the cross-linking step to preserve the electrostatically assembled PEI layer and to sustain the in situ interfacial environment within the ED system. Under these conditions, GA diffused to the membrane surface and reacted with the amine groups of PEI, forming a covalently cross-linked interfacial network.

#### 2.2.3. PSS Deposition

During the electro-assisted modification process, the anode was placed on the concentrate chamber side, while the cathode was placed on the DC. A direct current was applied, generating an electric field from the anode to the cathode across the membrane. PSS solution (0.31 g·L^−1^ in 25.7 g·L^−1^ NaCl) was fed into the DC, with 25.7 g·L^−1^ NaCl solution in CC. Positive-current ED was performed at 5 mA·cm^−2^ for 2.5 h at room temperature to deposit the negatively charged PSS layer via electrostatic attraction to the positively charged PEI–GA interfacial layer. The resultant CEM was denoted as PGP.

#### 2.2.4. Repetition of PEI-GA Coating

**Figure 1:**
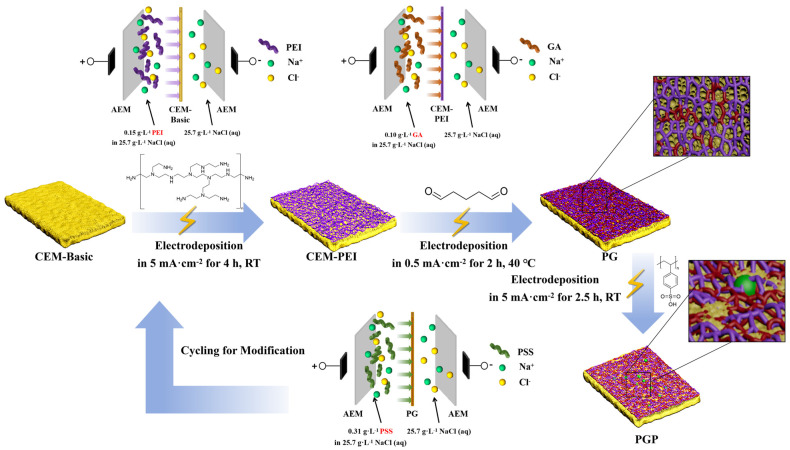
Route for the modification process of the CEM and the representation for structure.

To enhance the stability of the PSS layer, an additional PEI–GA coating cycle was applied as a capping layer on top of the PSS-modified membrane. Specifically, after completion of the PEI–GA–PSS modification sequence, the membrane was subjected again to PEI electro-assisted deposition followed by GA cross-linking, forming a PEI–GA capping layer over the PSS layer. The overall modification route and the corresponding interfacial layer architecture are schematically illustrated in Figure 1.

Furthermore, membranes subjected to two sequential PEI-GA coating cycles with different PEI concentrations were prepared and denoted as PGP-2 and PGP-2′. The number “2” indicates the second PEI-GA coating cycle, while the prime symbol (′) denotes a reversed order of PEI concentration (0.15 g·L^−1^ and 0.10 g·L^−1^, prepared in 25.7 g·L^−1^ NaCl solution) during the second PEI deposition step.

### 2.3. Material Characterization

#### 2.3.1. Characterization of Surface Morphology

The physicochemical properties of the pristine and modified CEMs were characterized by a series of analytical techniques. The surface chemical compositions were analyzed by X-ray photoelectron spectroscopy (Kratos Analytical, Manchester, UK), and the chemical structures were identified using Fourier-transform infrared spectroscopy (ATR-FTIR, Nicolet 6700, Thermo Fisher Scientific, Madison, WI, USA). Comprehensive analytical details for XRD and FTIR can be found in Supporting Information.

#### 2.3.2. Water Contact Angle

The surface hydrophobicity of unmodified and modified membranes was determined by water contact angle measurements using an optical tensiometer (OCA50AF, Dataphysics Instruments GmbH, Filderstadt, Germany). The dried membranes were fixed on a glass slide before the measurement. Measurements were performed four times with 4 µL DI water droplets for each membrane.

#### 2.3.3. Surface Area Resistance

The surface resistance of the membranes was measured using a multichannel electrochemical synthesizer (PARSTAT MC, Ametek, Berwyn, PA, USA). Before testing, each membrane was equilibrated in 0.5 M NaCl. The hydrated membrane was then positioned between two custom-made compartments, both filled with 0.5 M NaCl to maintain a uniform ionic environment during the measurement [44].

An alternating current signal was applied, and the total resistance (*r_t_*)—consisting of the membrane resistance (*R*) and the solution resistance (*r_s_*)—was obtained from the high-frequency intercept of the Nyquist plot on the real (Z) axis. The intrinsic membrane resistance (*R*) was calculated by subtracting the solution resistance and normalizing by the effective membrane area, as expressed in Equation (1).

(1) $$R={(r}_{t}-r_{s})\;\times \;A$$

#### 2.3.4. Limiting Current Density Measurement

The limiting current density (LCD) of the CEMs was determined using a four-compartment ED cell under steady-state conditions. Each compartment was filled with 0.05 M NaCl solution, and a pair of platinum electrodes was placed in the anode and cathode chambers of the ED cell and connected to a DC power supply to provide the driving electric field. The applied cell voltage was monitored directly from the power supply during operation, and no additional potential-sensing electrodes were used. The effective membrane area was 7.625 cm^2^. The current-voltage (I–V) characteristics were obtained by gradually increasing the applied current density from 0 to 40 mA·cm^−2^. The current was increased stepwise with a constant increment of 0.01 A using the power supply, corresponding to a fixed increment in current density after normalization by the effective membrane area. The LCD was identified from the breakpoint in the I–V curve, where the slope sharply increased due to concentration polarization. All experiments were performed at room temperature (25 ± 1 °C) under continuous stirring to minimize boundary layer effects.

#### 2.3.5. Evaluation of Separation of Li^+^/Mg^2+^ by ED

**Figure 2:**
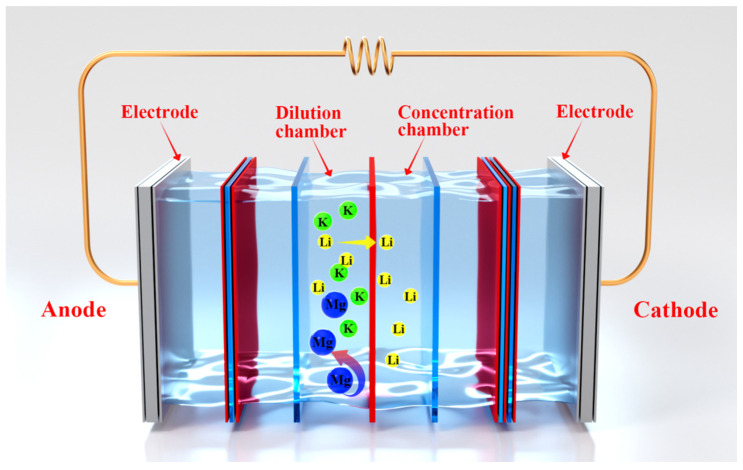
Schematic diagram of the configuration of ED and the process of ion separation. (Schematic diagram of the ED configuration and the ion separation process. The red membrane represents the CEM, while the blue membrane represents AEM. The green, blue, and yellow spheres represent potassium ions (K+), magnesium ions (Mg2^+^), and lithium ions (Li^+^) in the solution, respectively. DC was fed with the prepared brine solution, and CC was initially filled with DI water, and the electrode chambers were filled with a Na_2_SO_4_ solution. For clarity of illustration, the central compartments are intentionally drawn thicker than their actual dimensions).

The ion selectivity of the CEMs was evaluated using a four-compartment ED cell consisting of one DC, one CC, and two electrode chambers in Figure 2. The ED stack consisted of five repeating units, each of which included a monovalent selective CEM in series with an AEM. The system was divided into an electrode chamber, DC, and CC, used for reserving the electrode solution, brine feed, and DI water, respectively. The feed solution in the DC consisted of salt lake brine diluted four times. This dilution was applied to reduce the overall ionic strength of the brine, thereby mitigating severe concentration polarization and excessive voltage increase during ED operation, while enabling stable and reproducible evaluation of membrane separation performance. The tanks were connected to the membrane stack through submersible pumps. The electrode tank was filled with 0.5 L of a 30 g·L^−1^ Na_2_SO_4_ solution. The electrode chambers were filled with 0.5 L of a 30 g·L^−1^ Na_2_SO_4_ solution. The DC was filled with 1.0 L of the prepared brine feed, while the CC was filled with 1.0 L of DI water. The effective surface area of each membrane was 189 cm^2^, and 0.7 mm spacers were used to separate each membrane. A constant flow rate of 40 L/h was maintained.

The ED separation was performed in the forward-current mode at a constant current density of 20 mA·cm^−2^ for 1.5 h or 10 h. During operation, the pH, conductivity, and volume of each compartment were monitored. Samples were collected periodically and analyzed for the concentration of Li^+^ and Mg^2+^. The concentrations of Li^+^ and Mg^2+^ in the DC were determined at various intervals using inductively coupled plasma optical emission spectrometer (IC6200, Anhui Wanyi Science and Technology Co., Ltd., Hefei, China).

The ion flux (*J_i_*, mol·m^–2^·s^–1^) of species *i* through the membrane was determined according to Equation (2) [22]:

(2) $$Ji=\frac{V}{A}\times \frac{\Delta Ci}{\Delta t}$$

where *V* represents the volume of feed liquid (in cm^3^, *V* = 1 L), Δ*C_i_*/Δ*t* represents the average rate of change of ion concentration in DC over a sampling interval of 30 min., and *A* (in m^2^, *A* = 189 cm^2^) is the effective membrane area.

Subsequently, the cation perm-selectivity between Li^+^ and Mg^2+^ of the CEMs was determined utilizing Equation (3):

(3) $$P_{Li^{+}/Mg^{2+}}=\frac{J_{Li}C_{Mg}}{J_{Mg}C_{Li}}$$

where *J_i_* represents the mass transfer flux of ions (in mol·cm^−2^·s^−1^), *C_Mg_* (in mol·L^−1^) is the concentration of magnesium ions, and *C_Li_* (in mol·L^−1^) is the concentration of lithium ions.

## 3. Results and Discussion

### 3.1. Surface Structure Analysis

**Figure 3:**
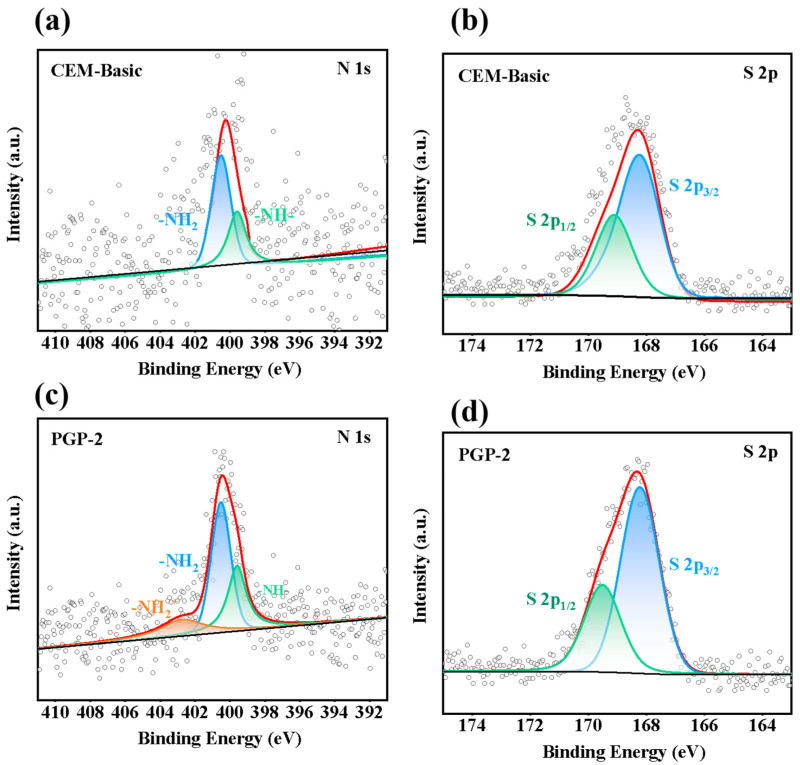
The XPS spectra of CEM-Basic (**a**) N 1s, (**b**) S 2p; the XPS spectra of PGP-2 (**c**) N 1s, (**d**) S 2p. The black solid line represents the experimental spectrum, colored lines correspond to the fitted component peaks, the red solid line indicates the overall fitting curve. The open circles represent the raw data points.

In the XPS analysis of CEM-Basic and PGP-2, a comparison of the S 2p spectra in Figure 3a,c reveals a significant enhancement of the doublet peaks at approximately 168.3 eV and 169.5 eV. This increase can be attributed not only to the inherent sulfonate groups of the base membrane but also to the effective incorporation of PSS, confirming that PSS has been successfully embedded into the surface multilayer structure through the stepwise, current-reversal-driven layer-by-layer assembly process. Meanwhile, the N 1s spectrum in Figure 3c displays three characteristic peaks at approximately 399.0 eV (imine-like species), 400.0 eV (neutral amine), and 401.5 eV (protonated amine). These peaks jointly verify the successful electro-deposition of PEI and its covalent cross-linking with GA, forming a stable framework for the interfacial layer. The imine-like species indicate a Schiff-base reaction between PEI and GA, leading to a covalently cross-linked structure. The –NH– originates from the unreacted amino groups of PEI. The –NH_2_^+^ at 401.5 eV, which is absent in CEM-Basic, suggests that during the electro-deposition process, local pH variations, or electrostatic interactions with sulfonate groups impart a partial positive charge on the membrane surface.

Combined with Figure S2, the XPS results reveal a clear and systematic evolution of surface composition during the stepwise modification process. Compared with the pristine CEM, the emergence of N 1s signals after PEI treatment confirms the successful deposition of a nitrogen-containing layer. Subsequent glutaraldehyde treatment leads to noticeable changes in the N 1s spectral features while maintaining the overall nitrogen signal, indicating stabilization, and restructuring of the deposited layer rather than its removal.

In the in-situ electro-assembly process, current reversal induces local electric-field changes at the membrane interface, promoting the association of PEI amino groups (-NH_2_/-NH-) with H^+^ in the solution under the applied electric field. This enhances the charge density of PEI and provides stronger positive-charge sites for the subsequent electrostatic adsorption of PSS. Moreover, during the in-situ electro-assembly process, the positively charged protonated PEI strongly adsorbs with the sulfonate groups (-SO_3_-) of PSS via electrostatic interactions, which also “compresses” the conformation of the PSS chains, causing them to pack more tightly against the PEI layer. This electro-assisted densification ultimately leads to the formation of a thicker PSS layer on the membrane surface, a result directly reflected in the significant enhancement of the S 2p signal intensity. Consequently, a multilayer interface characterized by high charge density and low defectivity is constructed, providing precisely tailored ion-transport pathways for the subsequent selective separation of lithium.

The FTIR images in Figure S4 further confirmed the successful modification of PGP-2. As shown in Figure S4, stronger absorption peaks appeared at ~1600 cm^−1^ (C = N stretching), which can be attributed to the cross-linking reaction between the amine groups of PEI and the aldehyde groups of GA via the Schiff-base reaction. The presence of sulfonate groups from PSS is evidenced by the symmetric and asymmetric S = O stretching vibrations around 1180 cm^−1^, respectively. Compared to the pristine CEM, the enhanced intensity of these sulfonate-related peaks, together with the emergence of the imine peak, directly verifies the incorporation of PSS and the formation of a cross-linked PEI network.

### 3.2. Physicochemical Properties

**Figure 4:**
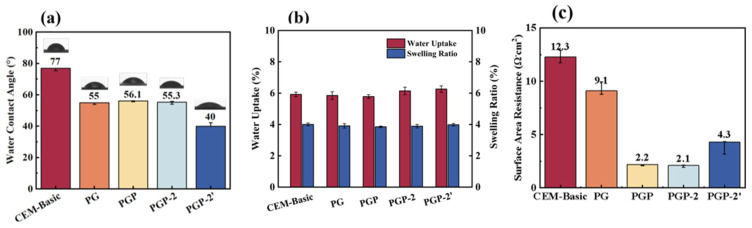
(**a**) The WCAs of the prepared CEMs; (**b**) WU and SR of the prepared CEMs; (**c**) the surface area resistance of the prepared CEMs.

The surface wettability of the pristine and modified membranes was evaluated by water contact angle (WCA), and the results are presented in Figure 4a. The CEM-Basic exhibited the highest WCA of 77°, indicating its relatively hydrophobic nature. The WCA of PG, which was deposited by PEI and GA, decreased significantly to 55°, reflecting the combined contribution of hydrophilic amine groups from PEI and polar functionalities introduced by GA, as well as the altered interfacial environment after cross-linking. When PSS was further incorporated, the WCA of PGP showed a slight increase to 56.1°, which could be attributed to the partial coverage of sulfonic groups by the subsequent PEI layer. In the case of the asymmetric modification, where different sequences of deposition of PEI were applied, the WCA of PGP-2 remained at a comparable level to 55.3°, suggesting that the wettability was mainly governed by the interplay between exposed -SO_3_- groups of PSS and –NH_2_ groups of PEI. Notably, PGP-2′ exhibited the lowest WCA of 40°, demonstrating more effective exposure of sulfonic acid groups and the synergistic effect of surface functional groups and the synergistic effect of surface functional groups resulting from the second PEI–GA treatment. The increased hydrophilicity of PGP-2′ is expected to facilitate ion transport during ED by promoting the formation of a stable hydration layer at the membrane–solution interface, thereby reducing interfacial transport resistance. Notably, such a highly hydrated interface favors the transport of monovalent Li^+^ with more labile hydration shells, while the migration of divalent Mg^2+^, which possess higher hydration energy and stronger hydration shells, remains hindered. This asymmetric effect contributes to the enhanced Li^+^/Mg^2+^ selectivity observed for PGP-2′ in the performance tests.

The area resistance of the pristine and modified membranes was measured to evaluate the effect of surface modification on ionic transport behavior [21]. To verify the reliability of the measured membrane resistance, electrochemical impedance spectroscopy (EIS) measurements were repeated, and a representative Nyquist plot is provided in the Supporting Information (Figure S5). As shown in Figure 4c, CEM-Basic exhibited the highest resistance of 12.3 Ω·cm^2^, which is consistent with its relatively hydrophobic surface and limited ion conductivity. In contrast, all modified membranes exhibited lower effective area resistance than the pristine membrane. This reduction does not arise from a decrease in the intrinsic bulk resistance of the membrane matrix, but rather from the introduction of a highly hydrated and ion-conductive interfacial layer, which mitigates interfacial transport barriers and improves ion continuity at the membrane–solution interface. As a result, the overall effective resistance governing ion migration during ED is reduced despite the presence of an additional surface layer. However, PGP-2 and PGP-2′ exhibited slightly increased resistance compared with PGP, which can be attributed to the formation of additional organic layers that hinder ion migration, but also maintained a relatively low resistance compared with CEM-Basic. It indicated that the multilayered PEI-GA-PSS modification preserved sufficient ionic channels while enhancing Li^+^/Mg^2+^ selectivity. Overall, the resistance results confirm that the modification strategy not only enhanced hydrophilicity but also significantly improved the ion transport properties of the membranes, which is beneficial for reducing energy consumption in ED.

**Figure 5:**
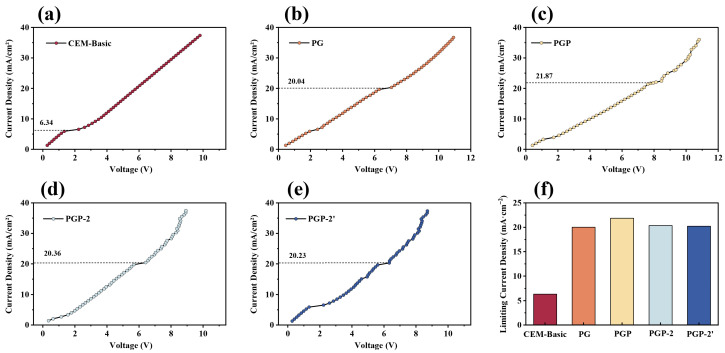
Voltage-current density curves of (**a**) CEM-Basic; (**b**) PG; (**c**) PGP; (**d**) PGP-2; (**e**) PGP-2′; and (**f**) limiting current density.

As shown in Figure 5, all membranes exhibited the typical three-stage behavior of IEMs, including the ohmic region, the limiting current plateau, and the over limiting region. CEM-Basic displayed the lowest limiting current density, indicating strong concentration polarization at the membrane–solution interface [47]. In contrast, the modified membranes (PG, PGP, PGP-2, and PGP-2′) display a systematic rightward shift of the transition point from the ohmic to the limiting region, indicating a progressive increase in limiting current density. This enhancement can be attributed to the introduction of hydrophilic and charged groups (-NH_2_ and -SO_3_-) wettability and ion transport channels. These functional groups not only improve surface hydrophilicity, thereby facilitating WU and reducing surface area resistance, but also establish a continuous, charge-enriched pathway that promotes counter-ion migration and mitigates concentration polarization. Among the modified series, PGP-2′ exhibits a slightly higher LCD compared to the other modified membranes, which is consistent with its optimized bilayer architecture. The secondary PEI deposition followed by GA cross-linking further densifies the interfacial layer while maintaining sufficient charge accessibility. As a result, efficient ion transport can be sustained without introducing pronounced mass-transfer limitations, enabling a higher tolerance to elevated current densities. This behavior is advantageous for maintaining stable electro-driven separation under practical operating conditions.

Notably, the I–V curves of the surface-modified membranes exhibit two plateau regions, in contrast to the single limiting current plateau observed for the pristine CEM. The first plateau appearing at lower current density is attributed to ion transport limitation within the surface-modified layer, where enhanced charge density and interfacial regulation induce an earlier onset of local concentration polarization. As the applied current further increases, ion transport through the bulk membrane and adjacent solution layers becomes the dominant limiting factor, giving rise to a second plateau corresponding to the classical diffusion-controlled limiting current. The relative positions of these two plateaus depend on the structure and hydration state of the surface layer, explaining the differences observed among PG, PGP, PGP-2, and PGP-2′ membranes.

### 3.3. The Selectivity of Li^+^/Mg^2+^ and Ion Transport Properties

**Figure 6:**
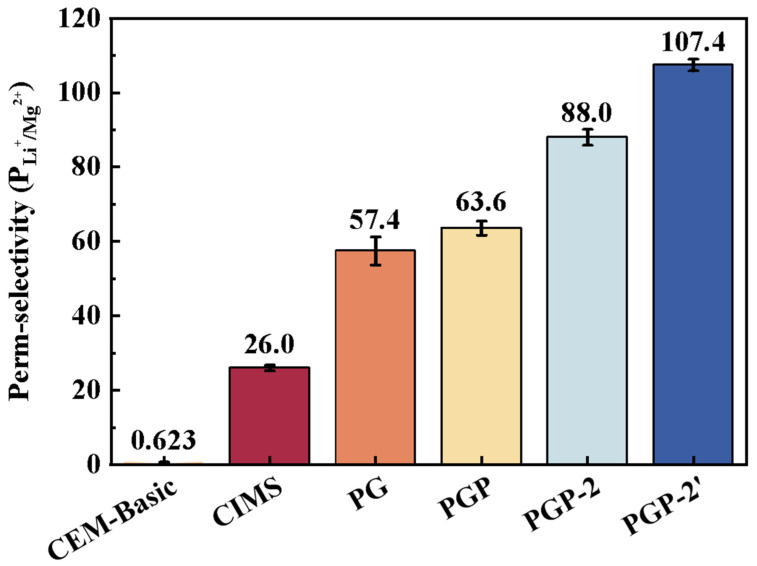
Comparison of Li^+^/Mg^2+^ perm-selectivity for commercial and prepared membranes in ED. (The perm-selectivity values are dimensionless and were calculated based on ion transport rates. The experiments were conducted using four-times-diluted natural brine collected from the Dongtai Salt Lake (Qinghai, China)).

The Li^+^/Mg^2+^ selectivities of the prepared CEMs and CEM-Basic were systematically investigated in ED process, and the results are summarized in Figure 6. The CEM-Basic exhibited almost no discrimination between divalent and monovalent cations, with a selectivity of only 0.62. After modification with PEI and GA, the selectivity of PG increased sharply to 57.4, which is far beyond the commercial selective cation exchange membrane CIMS. It showed that the introduction of polyamine layers, stabilized by covalent cross-linking, creates electrostatic barriers that preferentially hinder Mg^2+^ migration while allowing Li^+^ transport. In addition, the addition of PSS further improves the selectivity, which further densifies the interfacial layer through electrostatic complexation with the positively charged PEI layer. This dense surface layer creates a more stringent steric hindrance and diffusion barrier. Due to its larger hydrated ionic radius compared to Li^+^, Mg^2+^ experiences greater spatial resistance when passing through this dense layer, leading to a further reduction in its migration rate. The slight hydrophobicity introduced by PSS may work in concert with electrostatic repulsion and steric hindrance, establishing an additional “dehydration-transport” energy barrier that further differentiates between Li^+^ and Mg^2+^. Simultaneously, as a strong polyelectrolyte, PSS ionizes in solution via its sulfonate groups (-SO_3_-), introducing a high density of fixed negative charges to the membrane surface. This effect is indirectly supported by the ion exchange capacity (IEC) measurements, which show that the overall IEC of the modified membranes is maintained within a comparable range, indicating that the additional negative charges are mainly localized at the membrane interface rather than uniformly distributed throughout the membrane bulk (Table S3).

The selectivity of PGP-2 is 88.0, indicating that bilayer assembly improved structural stability. The high charge density PSS layer is “locked” and protected by the outermost PEI-GA cross-linked network. Interestingly, reversing the PEI concentration sequence in PGP-2′ yielded the highest selectivity *P_Li_*^+^_/_*_Mg_*^2+^ = 107.4. This superior performance can be attributed to the optimized balance between surface charge density and structural compactness, where the outer PEI-GA layer provides a strong electrostatic barrier and the inner PSS layer enhances electrostatic repulsion toward divalent Mg^2+^ while maintaining hydrated transport channels accessible to monovalent Li^+^. These results clearly demonstrate that the sequence and concentration of modifying agents play a decisive role in tuning ion transport. The optimized PGP-2′ configuration exhibits excellent potential for selective lithium recovery from brines, outperforming both commercial benchmarks and other laboratory-prepared membranes. Based on these results in model solutions, the modified membranes were further evaluated using real salt lake brine.

The constructed multilayer structure provides synergistic effects arising from surface charge modulation, electrostatic repulsion, and structural screening. Compared with conventional coating methods, the electric-field-driven initial deposition of PEI enables the formation of a more uniform and firmly anchored base layer. This optimized substrate promotes the subsequent adsorption of PSS, resulting in a more homogeneous distribution and higher stability of the introduced charged sites. Consequently, performance fluctuations caused by uneven charge distribution or detachment under high-salinity separation conditions are effectively mitigated. The subsequent layer-by-layer self-assembly between the negatively charged PSS and the positively charged PEI layer via strong electrostatic interactions significantly reduces the free volume within the interfacial region and increases its cross-linking density. This compact structure is further locked in place by covalent cross-linking with GA.

WCAs indicate that the incorporation of PSS moderately increases the surface hydrophobicity of the membrane. While this might be considered unfavorable in conventional understanding, it plays a crucial and beneficial role in the present system by enabling more efficient transport of Li^+^ compared to Mg^2+^. Although Li^+^ exhibits a relatively high hydration energy among monovalent cations, its hydration energy is significantly lower than that of Mg^2+^. This is because Mg^2+^ possesses an extremely high hydration energy and a strongly bound hydration shell with slow water-exchange kinetics. Therefore, a moderately hydrophobic interface essentially causes Mg^2+^ to be “retarded” at the interface due to its high dehydration energy barrier, whereas Li^+^, with a more labile hydration shell and faster water-exchange dynamics, can more readily undergo partial dehydration and proceed through the membrane, ultimately leading to the superior Li^+^/Mg^2+^ selectivity observed.

**Figure 7:**
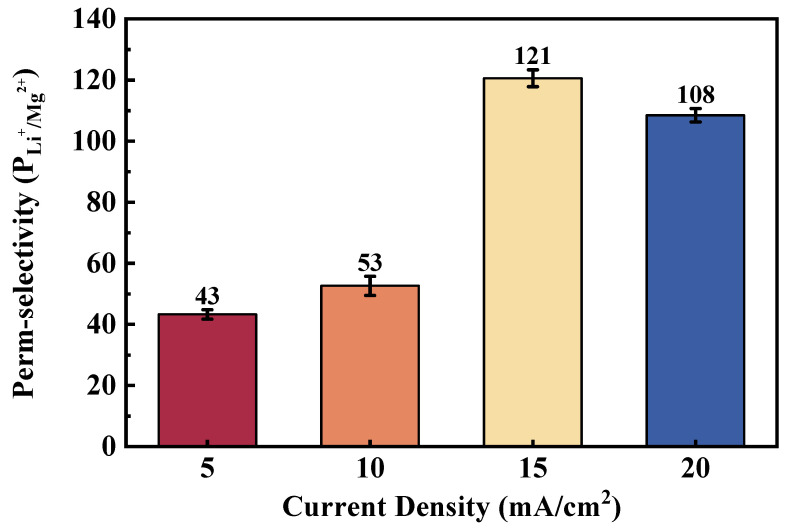
Effect of current density on Li^+^/Mg^2+^ selectivity of the prepared membranes in ED.

The influence of applied current density on the Li^+^/Mg^2+^ separation performance was evaluated within the range of 5–20 mA·cm^−2^. As shown in Figure 7, the selectivity exhibited a notable non-monotonic dependence on the current density, effect of applied current density on Li^+^/Mg^2+^ selectivity of the PGP-2′ membrane during ED. Experiments were conducted using four-times-diluted natural brine collected from the Dongtai Salt Lake (Qinghai, China). Specifically, the selectivity increased from 43 at 5 mA·cm^−2^ to 121 at 15 mA·cm^−2^, reaching a maximum, and then decreased slightly to 108 at 20 mA·cm^−2^. As the current density increases to 10 and 15 mA·cm^−2^, the enhanced electric field provides a stronger directional force that preferentially assists Li^+^ transport. The key mechanism is the electric-field-assisted partial dehydration at the membrane interface. Li^+^, with its smaller hydrated radius and faster water-exchange kinetics, can more readily shed its hydration shell under this increased field strength, facilitating its entry into and transport through the selective layer. In contrast, Mg^2+^, with its stronger electrostatic attraction to the negatively charged surface groups, experiences a proportionally greater energy barrier. This leads to a significant divergence in transport rates, maximizing selectivity at 15 mA·cm^−2^.

However, when the current density further increases beyond 15 mA·cm^−2^, enhanced concentration polarization and interfacial transport limitations become more pronounced, disturbing the electric double layer and reducing ion discrimination capability. The primary factor is likely intensified concentration polarization at the membrane-solution interface. At high current densities, although the bulk cation concentration in DC remains high (Mg^2+^ > 1 M), rapid ion transport can induce pronounced ion depletion in the immediate vicinity of the membrane surface. This localized depletion distorts the interfacial ionic environment and perturbs the structure of the electrical double layer, thereby weakening the Donnan equilibrium established at the membrane–solution interface. As a consequence, the effectiveness of Donnan exclusion is reduced, and ion transport becomes increasingly governed by interfacial transport limitations rather than by equilibrium electrostatic exclusion, which may ultimately lead to a diminished selectivity under high-current conditions. Furthermore, the increased voltage may induce minor water dissociation, altering the local pH and potentially affecting the charge state or swelling behavior of the polyelectrolyte layers. These combined effects disrupt the delicate balance of charge repulsion and size sieving, leading to a reduced ability to discriminate between Li^+^ and Mg^2+^.

In this study, the trend confirms that optimizing the applied current density is essential to balance ion mobility, hydration effects, and interfacial charge interactions for achieving high Li^+^/Mg^2+^ selectivity.

To evaluate the practical applicability of the PEI–GA–PSS–PEI–GA modified cation-exchange membrane, ED experiments were conducted using diluted salt lake brine as feed. Compared with model solutions, the natural brine matrix presents more complex challenges due to the coexistence of high Mg^2+^/Li^+^ ratio, competitive monovalent ions (Na^+^, K^+^), and divalent/trivalent impurities (Ca^2+^, Sr^2+^, etc.), which may strongly affect ion selectivity and long-term stability of the modified layer.

**Figure 8:**
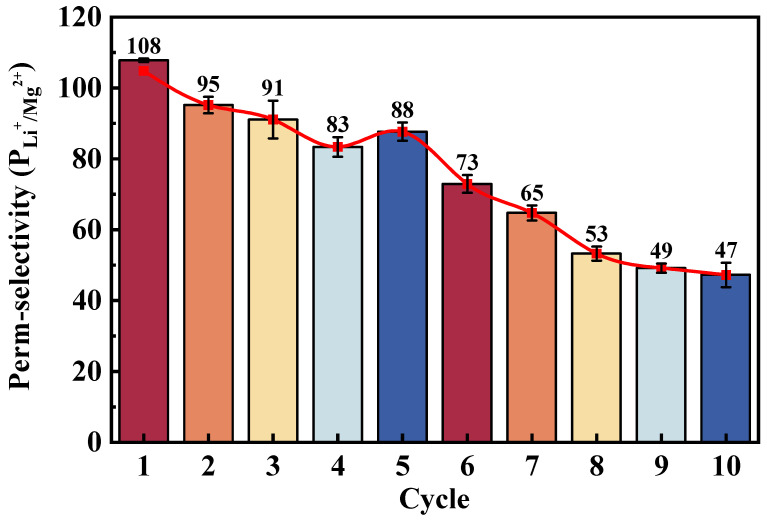
Cycling stability of Li^+^/Mg^2+^ selectivity for pristine and modified membranes over 10 ED cycles.

As shown in Figure 8**,** the PGP-2′ membrane over 10 consecutive ED cycles. Experiments were carried out using four-times-diluted natural brine collected from the Dongtai Salt Lake (Qinghai, China). One ED cycle was defined as an operation period of 90 min at a constant current density of 20 mA·cm^−2^, after which the solutions were refreshed before the next cycle. The modified membrane exhibited *P_Li_*^+^_/_*_Mg_*^2+^ = 108 in the first cycle, which remained at 47 after 10 cycle continuous operation. This selectivity was markedly superior to that of commercial monovalent-selective CEMs, under identical conditions. The higher selectivity is attributed to the synergistic effect of the cross-linked PEI network and the PSS-induced electrostatic exclusion, which together create a hybrid interface combining adsorption selectivity and Donnan-based repulsion.

In terms of long-term stability, the gradual decline in selectivity may be related to structural rearrangement of the multilayer coating or partial loss of weakly adsorbed PSS chains under electric field stress. Nevertheless, the membrane maintained better stability than commercial counterparts, suggesting that the multilayer modification strategy provides both enhanced initial selectivity and practical durability.

A comparative summary of recently reported Li^+^/Mg^2+^ separation performances achieved by different membrane systems is provided in Table S4, serving as a benchmark for evaluating the results obtained in this study. These results highlight the potential of the PEI–GA–PSS–PEI–GA strategy for industrial lithium extraction from high Mg^2+^/Li^+^ brines. By further optimizing the modification process (e.g., tuning layer thickness, introducing secondary cross-linking, or incorporating hydrophilic additives), the long-term performance could be further improved, paving the way for scalable application in salt lake brine treatment.

## 4. Conclusions

In conclusion, this work demonstrates a practical in-situ electro-assembly strategy that effectively bridges the gap between lab-scale membrane design and potential industrial application. The core innovation is an integrated online process for modifying cation exchange membranes directly within the ED stack, bypassing the need for disassembly. This operational breakthrough is achieved through a methodological advance—using programmed current reversal to precisely control the sequential deposition and cross-linking of polyelectrolytes, forming a chemically and structurally integrated composite interface.

The resulting membranes achieve high Li^+^/Mg^2+^ selectivity, attributed to the synergistic combination of size exclusion and enhanced electrostatic repulsion engineered by the tailored interfacial layer. Under the tested conditions, the membranes maintain acceptable Li^+^/Mg^2+^ selectivity over repeated ED cycles in real salt lake brine, demonstrating the short-term operational stability of the in-situ formed interface. This study establishes a versatile and scalable platform for the functionalization of membranes directly within their operational units, offering a promising path for industrial lithium recovery and other precision separations.

## Data Availability

The original contributions presented in this study are included in the article. Further inquiries can be directed to the corresponding authors.

## Supplementary Materials

The following supporting information can be downloaded at: https://www.mdpi.com/article/10.3390/membranes16030087/s1, Figure S1: The experimental device used in this work; Figure S2: High-resolution XPS spectra of CEM–PEI and PG membranes; Figure S3: The general XPS survey; Figure S4: ATR-FTIR spectra of the prepared membranes; Figure S5: Representative Nyquist plot obtained from AC impedance measurements for the determination of membrane resistance; Figure S6: pH-dependent zeta-potential of pristine and modified membranes. (a) CEM-Basic (b) PG (c) PGP-2’; Table S1: Information related to membrane modification in this work; Table S2: Measured ion concentrations (Li^+^, Mg^2+^, B) in the Dongtai Salt Lake brine; Table S3: IEC of pristine and surface-modified CEMs; Table S4: Comparison of literatures [40,53,54,55,56,57,58,59,60,61,62,63,64,65,66,67,68].

## References

[1] Zhao Y., Wu M., Shen P., Uytterhoeven C., Mamrol N., Shen J., Gao C., Van der Bruggen B. (2021). Composite anti-scaling membrane made of interpenetrating networks of nanofibers for selective separation of lithium. J. Membr. Sci..

[2] Ding T., Zheng M., Peng S., Lin Y., Zhang X., Li M. (2023). Lithium extraction from salt lakes with different hydrochemical types in the Tibet Plateau. Geosci. Front..

[3] Lang J., Qi L., Luo Y., Wu H. (2017). High performance lithium metal anode: Progress and prospects. Energy Storage Mater..

[4] Ding B., Wang J., Fan Z., Chen S., Lin Q., Lu X., Dou H., Kumar Nanjundan A., Yushin G., Zhang X. (2020). Solid-state lithium–sulfur batteries: Advances, challenges and perspectives. Mater. Today.

[5] Xu T., Shehzad M.A., Yu D., Li Q., Wu B., Ren X., Ge L., Xu T. (2019). Highly Cation Permselective Metal–Organic Framework Membranes with Leaf-Like Morphology. ChemSusChem.

[6] Liu C., Li Y., Lin D., Hsu P.-C., Liu B., Yan G., Wu T., Cui Y., Chu S. (2020). Lithium Extraction from Seawater through Pulsed Electrochemical Intercalation. Joule.

[7] Zeng Y., Liao S., Wang K., Lv N., Luo J., Ye G., Yu X. (2022). Stable Phase Equilibria of Ternary Systems Li^+^,Rb^+^//SO_4_^2−^–H_2_O and Li^+^,Cs^+^//SO_4_^2−^–H_2_O at 273.2 K. ACS Omega.

[8] Guo Z.-Y., Wang J., Zhang P., Wang L., Lai Z., Ji Z.-Y. (2026). Lithium selective membranes for direct lithium extraction from complex brine. Prog. Mater. Sci..

[9] Zhang J., Xi W., Hu J., Han Q., Zhang Y., Huang S., Wang R., Wang H., Gong Y., Jin J. (2025). Optimizing lithium extraction from brines using fluoride-doped lithium titanate in hybrid capacitive deionization. Desalination.

[10] Zavahir S., Elmakki T., Gulied M., Ahmad Z., Al-Sulaiti L., Shon H.K., Chen Y., Park H., Batchelor B., Han D.S. (2021). A review on lithium recovery using electrochemical capturing systems. Desalination.

[11] Zhang Y., Wang L., Sun W., Hu Y., Tang H. (2020). Membrane technologies for Li^+^/Mg^2+^ separation from salt-lake brines and seawater: A comprehensive review. J. Ind. Eng. Chem..

[12] Deng X., Li J., Lai P., Kuang S., Liu J., Dai P., Hua H., Dong P., Zhang Y., Yang Y. (2023). Tailored interface composition improves the integrity of electrode/electrolyte interphases for high-voltage Ni-rich lithium metal batteries in a sulfolane-based electrolyte. Chem. Eng. J..

[13] Strathmann H. (2010). Electrodialysis, a mature technology with a multitude of new applications. Desalination.

[14] Sata T., Sata T., Yang W. (2002). Studies on cation-exchange membranes having permselectivity between cations in electrodialysis. J. Membr. Sci..

[15] Xu P., Capito M., Cath T.Y. (2013). Selective removal of arsenic and monovalent ions from brackish water reverse osmosis concentrate. J. Hazard. Mater..

[16] Kabay N., Arda M., Kurucaovali I., Ersoz E., Kahveci H., Can M., Dal S., Kopuzlu S., Haner M., Demircioglu M. (2003). Effect of feed characteristics on the separation performances of monovalent and divalent salts by electrodialysis. Desalination.

[17] Kabay N., İpek Ö., Kahveci H., Yüksel M. (2006). Effect of salt combination on separation of monovalent and divalent salts by electrodialysis. Desalination.

[18] Kabay N., Kahveci H., İpek Ö., Yüksel M. (2006). Separation of monovalent and divalent ions from ternary mixtures by electrodialysis. Desalination.

[19] Zhang Y., Paepen S., Pinoy L., Meesschaert B., Van der Bruggen B. (2012). Selectrodialysis: Fractionation of divalent ions from monovalent ions in a novel electrodialysis stack. Sep. Purif. Technol..

[20] Zhang Y., Van der Bruggen B., Pinoy L., Meesschaert B. (2009). Separation of nutrient ions and organic compounds from salts in RO concentrates by standard and monovalent selective ion-exchange membranes used in electrodialysis. J. Membr. Sci..

[21] Sata T. (2000). Studies on anion exchange membranes having permselectivity for specific anions in electrodialysis—Effect of hydrophilicity of anion exchange membranes on permselectivity of anions. J. Membr. Sci..

[22] Tekinalp Ö., Zimmermann P., Birger Byremo Solberg S., Stokke Burheim O., Deng L. (2023). Selective recovery of silver from copper impurities by electrodialysis: Tailoring monovalent selective cation exchange membranes by monomolecular layer deposition. Chem. Eng. J..

[23] Nitodas S.F., Das M., Shah R. (2022). Applications of Polymeric Membranes with Carbon Nanotubes: A Review. Membranes.

[24] Xing Z., Srinivasan M. (2023). Lithium recovery from spent lithium-ion batteries leachate by chelating agents facilitated electrodialysis. Chem. Eng. J..

[25] Wang J., Yue X., Wang P., Yu T., Du X., Hao X., Abudula A., Guan G. (2022). Electrochemical technologies for lithium recovery from liquid resources: A review. Renew. Sustain. Energy Rev..

[26] Ge M., Wei C., Fang T., Liu X. (2024). Molecular insight into the separation mechanism of crown Ether-Based channels for lithium Extraction. Sep. Purif. Technol..

[27] Wang W., Zhang Y., Yang X., Sun H., Wu Y., Shao L. (2023). Monovalent Cation Exchange Membranes with Janus Charged Structure for Ion Separation. Engineering.

[28] Ge L., Wu B., Li Q., Wang Y., Yu D., Wu L., Pan J., Miao J., Xu T. (2016). Electrodialysis with nanofiltration membrane (EDNF) for high-efficiency cations fractionation. J. Membr. Sci..

[29] Hou L., Wu B., Yu D., Wang S., Shehzad M.A., Fu R., Liu Z., Li Q., He Y., Afsar N.U. (2018). Asymmetric porous monovalent cation perm-selective membranes with an ultrathin polyamide selective layer for cations separation. J. Membr. Sci..

[30] Wang W., Liu R., Tan M., Sun H., Niu Q.J., Xu T., Nikonenko V., Zhang Y. (2019). Evaluation of the ideal selectivity and the performance of selectrodialysis by using TFC ion exchange membranes. J. Membr. Sci..

[31] Guo Y.-S., Weng X.-D., Wu B., Mi Y.-F., Zhu B.-K., Ji Y.-L., An Q.-F., Gao C.-J. (2019). Construction of nonfouling nanofiltration membrane via introducing uniformly tunable zwitterionic layer. J. Membr. Sci..

[32] Yan L., Yang X., Zhao Y., Wu Y., Motlhaletsi Moutloali R., Mamba B.B., Sorokin P., Shao L. (2022). Bio-inspired mineral-hydrogel hybrid coating on hydrophobic PVDF membrane boosting oil/water emulsion separation. Sep. Purif. Technol..

[33] Rijnaarts T., Reurink D.M., Radmanesh F., de Vos W.M., Nijmeijer K. (2019). Layer-by-layer coatings on ion exchange membranes: Effect of multilayer charge and hydration on monovalent ion selectivities. J. Membr. Sci..

[34] Cao R., Shi S., Duan F., Xu Y., Li Y., Cao H., Wang Y. (2023). In-situ construction of modified layer on the surface of anion exchange membrane to improve antifouling performance. Desalination.

[35] Cengiz H.Y., Konyali E., Müftüler A., Deligöz H. (2023). Investigating the effect of weak polyelectrolytes on the chemical stability and swelling recovery of multilayered coatings. Prog. Org. Coat..

[36] Xing K., Wang M., Pan B., Liang C., Li Y. (2025). Efficient Bicarbonate Electrolysis to Formate Enabled via Ionomer Surface Modification in Cation Exchange Membrane Electrolyzers. Angew. Chem. Int. Ed..

[37] Ge L., Wu B., Yu D., Mondal A.N., Hou L., Afsar N.U., Li Q., Xu T., Miao J., Xu T. (2017). Monovalent cation perm-selective membranes (MCPMs): New developments and perspectives. Chin. J. Chem. Eng..

[38] Abdu S., Martí-Calatayud M.-C., Wong J.E., García-Gabaldón M., Wessling M. (2014). Layer-by-Layer Modification of Cation Exchange Membranes Controls Ion Selectivity and Water Splitting. ACS Appl. Mater. Interfaces.

[39] Tekinalp O., Zimmermann P., Holdcroft S., Burheim O.S., Deng L. (2023). Cation Exchange Membranes and Process Optimizations in Electrodialysis for Selective Metal Separation: A Review. Membranes.

[40] Sun M., Mu C., Wang S., Bi J., Guo X., Wang S., Zhao Y. (2024). Monovalent cation exchange membranes prepared by Fe^3+^-assisted coupled surface modification of PEI-PPy for potassium extraction from simulated brine. Desalination.

[41] Wang W., Zhang Y., Li F., Chen Y., Mojallali Rostami S.M., Hosseini S.S., Shao L. (2022). Mussel-inspired polyphenol/polyethyleneimine assembled membranes with highly positive charged surface for unprecedented high cation perm-selectivity. J. Membr. Sci..

[42] Cheng C., Yaroshchuk A., Bruening M.L. (2013). Fundamentals of Selective Ion Transport through Multilayer Polyelectrolyte Membranes. Langmuir.

[43] Sahin S., Dykstra J.E., Zuilhof H., Zornitta R.L., de Smet L.C.P.M. (2020). Modification of Cation-Exchange Membranes with Polyelectrolyte Multilayers to Tune Ion Selectivity in Capacitive Deionization. ACS Appl. Mater. Interfaces.

[44] Wei J., Ma X., Yang F., Jia S., Wang Z. (2025). High-efficiency membrane for Mg^2+^/Li^+^ separation prepared via grafting symmetrical bis-quaternary ammonium salt. J. Membr. Sci..

[45] Zu S., Liu Q., Sun Z., Zhu Y., Meng Q., Liu Q., Liu H., Xing H., Yang L. (2025). Microphase-separated lithium ion sieve nanoporous composite membrane for lithium extraction from salt lake brine. Desalination.

[46] Xu J., Mu J., Yao Y., Xu Y., Liao J., Ruan H., Shen J. (2023). Ion Resource Recovery via Electrodialysis Fabricated with Poly(Arylene Ether Sulfone)-Based Anion Exchange Membrane in Organic Solvent System. Small.

[47] Ruan H., Gao S., Li Y., Yu S., Liao J., Ang E.H., Xu Y., Shen J. (2024). Optimization of the mass ratio of siloxane crosslinkers for poly(2,6-dimethyl-1,4-phenylene oxide) anion exchange membranes to improve acid enrichment by electrodialysis. J. Membr. Sci..

[48] Salmeron-Sanchez I., Asenjo-Pascual J., Avilés-Moreno J.R., Pérez-Flores J.C., Mauleón P., Ocón P. (2022). Chemical physics insight of PPy-based modified ion exchange membranes: A fundamental approach. J. Membr. Sci..

[49] Khoiruddin, Ariono D., Subagjo, Wenten I.G. (2017). Surface modification of ion-exchange membranes: Methods, characteristics, and performance. J. Appl. Polym. Sci..

[50] Li J., Yuan S., Wang J., Zhu J., Shen J., Van der Bruggen B. (2018). Mussel-inspired modification of ion exchange membrane for monovalent separation. J. Membr. Sci..

[51] Sata T. (1992). Properties of ion exchange membranes combined with conducting polymers anisotropically—I. emf generation by redox reactions across composite membrane. Electrochim. Acta.

[52] Gohil G.S., Binsu V.V., Shahi V.K. (2006). Preparation and characterization of mono-valent ion selective polypyrrole composite ion-exchange membranes. J. Membr. Sci..

[53] Rodriguez-Cruz S.E., Jockusch R.A., Williams E.R. (1999). Hydration energies and structures of alkaline earth metal ions, M^2+^(H_2_O)_n_, n = 5−7, M = Mg, Ca, Sr, and Ba. J. Am. Chem. Soc..

[54] Rempe S.B., Pratt L.R., Hummer G., Kress J.D., Martin R.L., Redondo A. (2000). The hydration number of Li^+^ in liquid water. J. Am. Chem. Soc..

[55] Nightingale E.R. (1959). Phenomenological theory of ion solvation. Effective radii of hydrated ions. J. Phys. Chem..

[56] Kingsbury R. (2025). A guide to ion separations for the global energy transition. Joule.

[57] Chen S., Mao C., Hu B., Zhang W., Deng H. (2022). Simultaneous improvement of flux and monovalent selectivity of multilayer polyelectrolyte membranes by ion-imprinting. Desalination.

[58] Mu T., Zhang Z., Guo L., Xu G., Liao J., Weng J., Shen J., Gao C. (2025). Quaternized poly (arylene piperidine) anion exchange membranes with enhanced alkaline stability and desalination performance. Desalination.

[59] Wang W., Wang C., Huang R., Hong G., Zhang Y., Zhang X., Shao L. (2025). Boosting lithium/magnesium separation performance of selective electrodialysis membranes regulated by enamine reaction. Water Res..

[60] Qian H., Xu G., Yang S., Ang E.H., Chen Q., Lin C., Liao J., Shen J. (2024). Advancing lithium–magnesium separation: Pioneering swelling-embedded cation exchange membranes based on sulfonated poly (ether ether ketone). ACS Appl. Mater. Interfaces.

[61] Zhang Q., Liu W., Wang X., Ding W., Han G., Liu S. (2025). Preparation and performance study of highly permeable and selective lithium-magnesium separation nanofiltration membranes modified by phospholipid interlayer. Sep. Purif. Technol..

[62] Yong M., Yang Y., Sun L., Tang M., Wang Z., Xing C., Hou J., Zheng M., Chui T.F.M., Li Z. (2024). Nanofiltration membranes for efficient lithium extraction from salt-lake brine: A critical review. ACS Environ. Au.

[63] Wang F., He K., Wang Z., Ma H., Shi F., Li Z., Zhou X., Wu Q., Acharya D., Doherty C.M. (2026). Asymmetric design of ion-transport channels in a polymeric membrane for lithium-ion sieving. J. Membr. Sci..

[64] Zhao G., Sun J., Tang G., Pan G., Yu H., Li Y., Zhang Y., Liu Y. (2024). Highly selective Mg^2+^/Li^+^ separation membranes prepared by surface grafting of a novel quaternary ammonium bromide. Sep. Purif. Technol..

[65] Seo D., Lee J., Kong S.R., Sim G., Park Y. (2025). Selective lithium extraction from salt-lake brine using LATP-incorporated cellulose membranes in electrically driven systems. J. Membr. Sci..

[66] Wang R., Alghanayem R., Lin S. (2023). Multipass nanofiltration for lithium separation with high selectivity and recovery. Environ. Sci. Technol..

[67] Koukoufilippou D., Liakos I.L., Pilatos G.I., Plakantonaki N., Banis A., Kanellopoulos N.K. (2024). Separation of Magnesium and Lithium Ions Utilizing Layer-by-Layer Polyelectrolyte Modification of Polyacrylonitrile Hollow Fiber Porous Membranes. Materials.

[68] Das S., Deng E., Tandel A.M., Singh S., Mowatt J.V., Lin H. (2026). Nanofiltration Membranes for Li^+^/Mg^2+^ Separation: Materials and Mechanisms. J. Polym. Sci..

