# Supplementary material for: Synergistic Interfacial Design of Cation Exchange Membranes via Sequential Electro-Assembly for High-Efficiency Lithium Separation
Source: Membranes (Basel). 2026 Feb 28;16(3):87. doi: 10.3390/membranes16030087 (PMC13027606; doi:10.3390/membranes16030087)
Supplement: Supplementary file 1 [file membranes-16-00087-s001.zip › membranes-4123221-supplementary.pdf]

Supporting Information

of

Synergistic interfacial design of cation exchange membranes via sequential electro-assembly for high-efficiency lithium separation

Zhibo Zhang [a,b,#], Geting Xu [a,b,#], Yangbo Qiu [c], Junbin Liao [a,b], Tong Mu [a,b], Wanji Zhou [d], Yunfang Gao [a,b,*], Jianquan Weng [a,b,*], Jiangnan Shen [a,b,*]

[a] College of Chemical Engineering, Zhejiang University of Technology, Hangzhou 310014, China.

[b] State Key Laboratory of Advanced Separation Membrane Materials, National Key Laboratory of Green Chemical Synthesis and Transformation Technology, Zhejiang University of Technology, Hangzhou 310014, China.

[c] Department of Civil Engineering, The University of Hong Kong, Pokfulam, Hong Kong, SAR 999077, China

[d] Salt Lake Chemical Engineering Research Complex, School of Chemical Engineering, Qinghai University, Xining 810016, Qinghai, China

* Corresponding Authors: Yunfang Gao(gaoyf@zjut.edu.cn) ;Jianquan Weng(wengjq@zjut.edu.cn); Jiangnan Shen (E-mail: shenjn@zjut.edu.cn).

[#] Equally contributed.

1. Materials and Reagents

Commercial cation exchange membranes (CEM, sulfonated type) were purchased from [Baichen China] and used as the base membranes without further pretreatment, unless otherwise specified. polyethyleneimine (PEI, molecular weight ≈ 25 kDa, purity ≥ 99%), glutaraldehyde (GA, 25 wt% aqueous solution), and poly(sodium 4-styrenesulfonate) (PSS, average molecular weight ≈ 70 kDa, purity ≥ 98%) were obtained from [Beijing Bailingwei Science and Technology Co., Ltd. China]. Sodium chloride (NaCl, analytical grade), All chemicals were used as received without additional purification.

The monovalent-selective cation exchange membrane (MCEM) used in this study is the CIMS membrane manufactured by Astom Corporation, Japan. The membrane has

a thickness of 0.15 mm, and a water uptake of approximately 28%. This commercial membrane was used as a benchmark for evaluating the separation performance of the modified membranes.

The feed solution for electrodialysis tests was prepared using either raw salt lake brine, provided by Brine from the Dongtai Salt Lake in Qinghai, China.

2. Membrane Modification Procedures

The modification process was carried out through a stepwise electro-assisted layer-by-layer (E-LbL) assembly.

Table S1. Information related to membrane modification in this work

Name	a			b	
	PEI (g/L)	GA (g/L)	PSS (g/L)	PEI (g/L)	GA (g/L)
CEM-Basic	/	/	/	/	/
PG	0.15	1	/	/	/
PGP	0.15	0.31	0.5	/	/
PGP-2	0.15	0.31	0.5	0.1	0.31
PGP-2’	0.1	0.31	0.5	0.15	0.31

a represents the first electrodeposition;

b represents the second electrodeposition.

The PEI solution (0.15 $g \cdot L^{-1}$), prepared in 25.7 $g \cdot L^{-1}$ NaCl, was used. Under these conditions, PEI is partially protonated and exist predominantly in a polycationic form, enabling electrostatic migration and deposition under an applied electric field.

The concentrations of PEI, GA, and PSS were selected based on commonly reported ranges in polyelectrolyte-assisted membrane modification studies, combined with preliminary experiments to ensure effective interfacial assembly without excessive layer densification. The concentration of NaCl was chosen to provide sufficient ionic strength for stable current-driven electro-assembly and to maintain consistent solution conductivity during the modification process.

Table S2. Measured ion concentrations (Li^+, Mg^{2+}, B) in the Dongtai Salt Lake brine

Element	Li^+	Mg^{2+}	B
Mass Fraction(wt%)	0.096	2.4	0.327

Concentration(mol/L)	0.166	1.1185	0.363
Hydration Energy (kJ/mol)	-519	-1920	/
Hydration Radius (Å)	3.40	4.28	/

Hydration energy and hydrated radius data are literature-derived parameters[1-4]. The brine is chloride-dominated; other anions (e.g., SO_4^{2-}) are present but not the focus of this study.

3. Characterization Methods

3.1 Water Contact Angle

The water contact angle (WCA) is an important reference indicator of membrane hydrophilicity, reflecting the contact state of water droplets on the membrane surface[5]. Prior to the measurement, the membranes were dried and fixed on a glass slide to ensure stable sample positioning and to obtain reproducible structural and chemical information. It should be noted that this characterization was performed to analyze the intrinsic membrane surface properties, whereas all separation and electrochemical performance tests were conducted with fully hydrated membranes. A water droplet with a volume of 1.0 mL was deposited onto the membrane surface using a micro-syringe, and the WCA at room temperature was measured using a contact angle measuring device (JY-PHa). For each sample, the static WCA was measured at least three different positions to evaluate the the hydrophilicity of the membrane surface.Surface resistance: Determined by a four-probe conductivity tester (RST-5000), and the area resistance ($\Omega\cdot cm^2$) was calculated accordingly.

3.2 Water Uptake and Swelling Ratio

The water uptake (WU) and swelling ratio (SR) of the CEMs were determined by measuring the weight and dimensional changes of the membranes in dry and wet states. Prior to measuring the WU and SR, membrane samples (1 cm × 4 cm) were thoroughly dried under vacuum at 60 °C for 24 h, and the mass (W_{dry}, g) and length (L_{dry}, cm) of the dry samples were measured. Subsequently, the samples were immersed in deionized water at 25 °C and 80 °C for 24 h to obtain fully hydrated AEM samples, and the weight (W_{wet}, g) and length (L_{wet}, cm) of the hydrated samples were

recorded. The water uptake and swelling ratio were calculated using Equations (1) and (2), respectively.

$$WU = \frac{W_{wet} \times W_{dry}}{W_{dry}} \quad (1)$$

$$SR = \frac{L_{wet} \times L_{dry}}{L_{dry}} \quad (2)$$

3.3 Ion Exchange Capacity

The ion exchange capacity (IEC) of CEM was determined using an acid-base titration method. Prior to measurement, membrane samples were thoroughly rinsed with DI water to remove residual salts and then dried in a vacuum oven at 60 ℃ until a constant weight was achieved. The dry weight of the membrane was recorded as m_{dry}.

To convert the membranes into the H^+ form, the dried samples were immersed in a 1.0 $mol \cdot L^{-1}$ HCl solution for 24 h, during which the acid solution was refreshed several times to ensure complete protonation of the ion-exchange sites. Afterward, the membranes were repeatedly washed with DI water until the washing solution reached near-neutral pH, indicating the removal of excess free acid.

The H^+-form membranes were then transferred into a known volume of 0.1 $mol \cdot L^{-1}$ NaCl solution and soaked for 24 h at room temperature to allow complete exchange of H^+ with Na^+. The released H^+ in the soaking solution were subsequently quantified by titration with a standardized NaOH solution using phenolphthalein as the indicator . All titrations were performed at least in triplicate to ensure reproducibility.

The IEC of the membranes was calculated according to Equation (3):

$$IEC = \frac{0.1 \times L_3 \times L_1 / L_2}{m_{dry}} \quad (3)$$

Where, m_{dry} denotes the mass of the dry membrane, L_1 represents the total volume of the soaking solution, L_2 is the volume of the aliquot withdrawn from the soaking solution, and L_3 corresponds to the volume of 0.05 $mol \cdot L^{-1}$ NaOH solution consumed

during titration.

Table S3. IEC of pristine and surface-modified CEMs

	**IEC mmol/g**
CEM-Basic	1.89
PG	1.96
PGP	1.62
PGP-2	1.58
PGP-2'	1.41

3.3 Surface Area Resistance

Electrochemical impedance spectroscopy (EIS) measurements were performed using a multichannel potentiostat (DH7002A) over a frequency range from 10^5 to 0.1 Hz. Before testing, each membrane was equilibrated in 0.5 M NaCl. The hydrated membrane was then positioned between two custom-made compartments, both filled with 0.5 M NaCl to maintain a uniform ionic environment during the measurement[6].

3.4 Limiting Current Density

The limiting current density of the ionic membrane was derived by testing the I-V characteristic curves of the CEMs. Before testing, each membrane was equilibrated in 0.5 M NaCl. Using a self-designed ED apparatus, the membrane sample and two pieces of AEM type II divided the apparatus into four compartments. Each side of the membrane sample was filled with a 0.05 M NaCl aqueous solution, while the compartment adjacent to the AEM type II contained a 0.3 M Na_2SO_4 aqueous solution. The initial current was set to 0.01 A and subsequently increased in steps of 0.01 A, corresponding to a constant increment in current density based on the effective membrane area.

3.5 Evaluation of Electrodialysis Performance

A four-compartment ED cell was employed to evaluate the ion separation performance of the pristine and modified CEMs. The ED stack consisted of a diluate chamber (DC),

a concentrate chamber (CC), and two electrode chambers, separated by alternating anion exchange membrane (AEM, Neosepta AMX) and the prepared CEMs[7]. The effective membrane area was 189 cm^2, and the electrodes were connected to a DC power supply to deliver a constant current density ranging from 5 to 20 $mA \cdot cm^{-2}$.

The applied current density and modification time were determined within a stable operational window identified from current-voltage measurements and preliminary tests, ensuring effective electro-assisted assembly without inducing severe concentration polarization or water splitting. All experiments were conducted at ambient temperature to avoid additional thermal effects and to reflect practical electrodialysis operating conditions.

During the ED process, the diluate chamber and concentrate chamber were filled with 4× diluted salt lake brine, while the electrode chambers were circulated with 30g/L Na_2SO_4 solution to maintain electrical conductivity and prevent electrode reactions.

Samples (100 μL) were withdrawn from both DC and CC at regular intervals to determine the Li^+ and Mg^{2+} concentrations using an ICP-OES The electrodialysis operation was conducted for 10 cycle, and the stack voltage was recorded simultaneously by a data acquisition system.

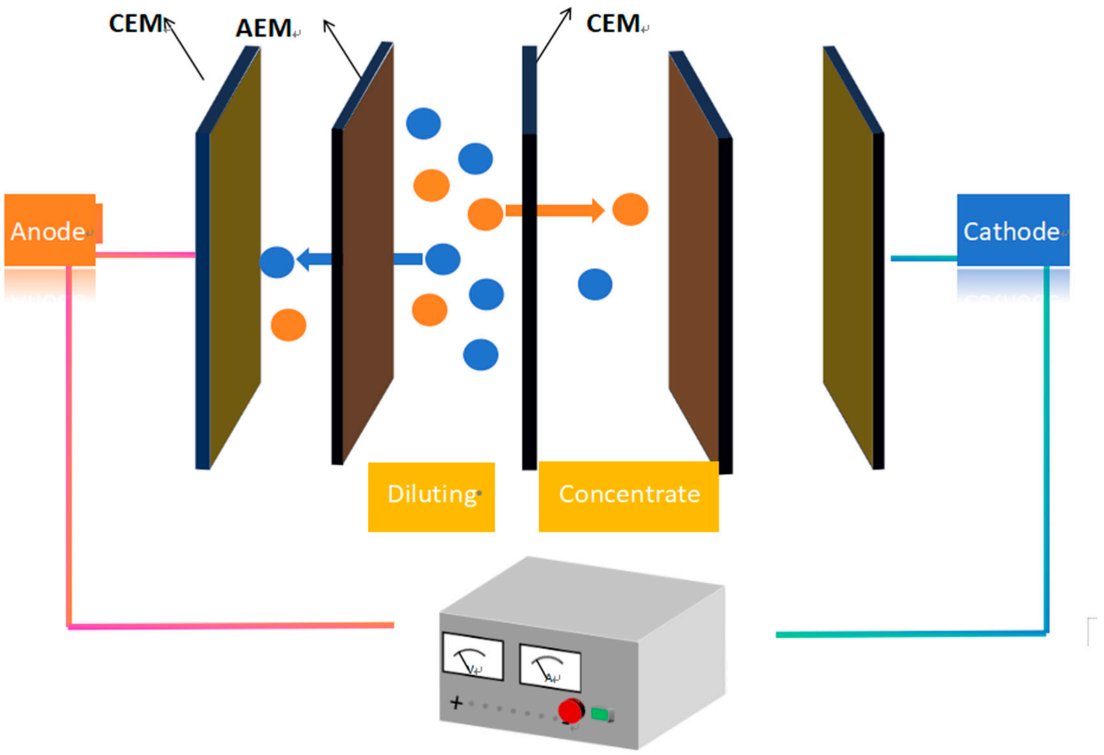

Figure S1. The experimental device used in this work

3.6 Surface Chemistry and Bonding Characterization

XPS measurements were performed using a Kratos Axis Ultra DLD spectrometer (Kratos Analytical, SHIMADZU, JAPAN). The instrument is equipped with a monochromatized aluminum X-ray source powered at 15 kV and 3 mA that delivers an X-ray beam of 300 × 700 μm^2. Charge compensation was obtained with the built-in charge neutralization system. The pass energy was set to 160 eV for the survey spectra and to 20 eV, 40 eV or 80 eV for the high-resolution spectra. The binding energies were calculated with respect to the C−(C, H) component (BE=284.8 eV) of the C1s peak, and a Shirley background subtraction was used.

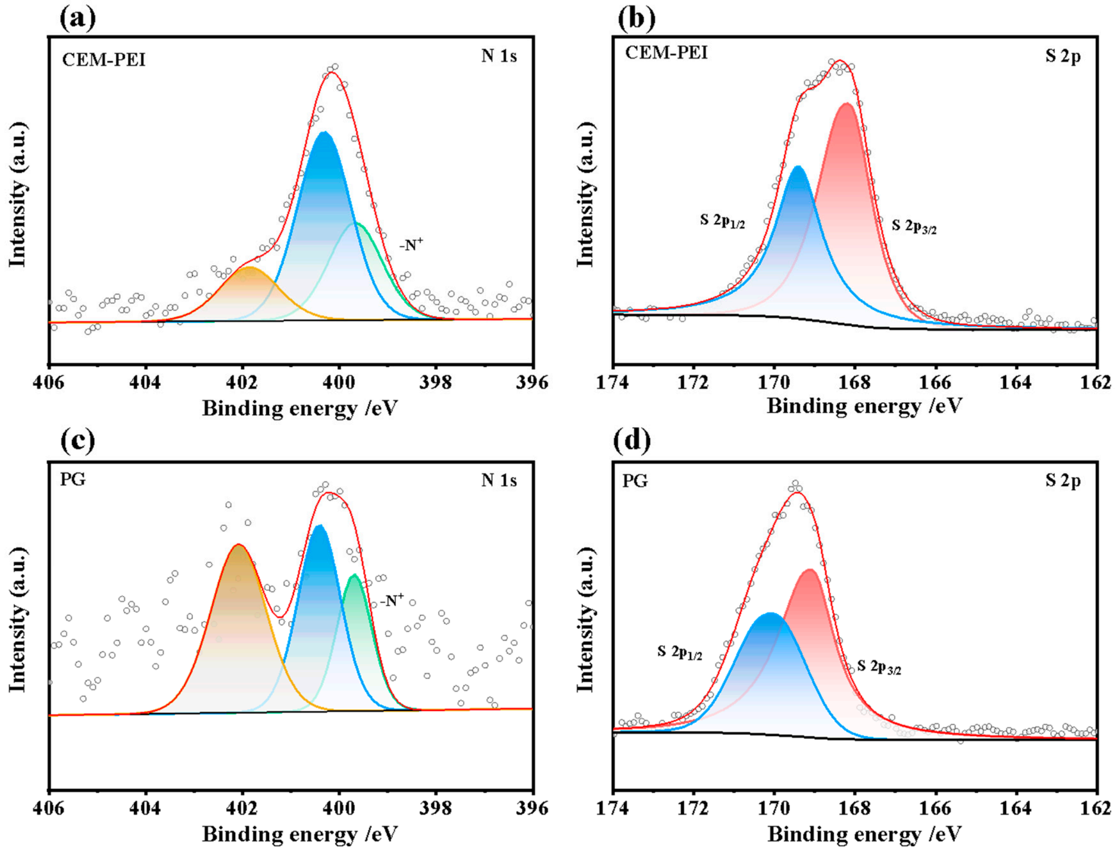

Figure S2. High-resolution XPS spectra of CEM–PEI and PG membranes

3.7 Zeta potential measurements

The surface charge properties of the membranes were characterized by zeta potential measurements using an electrokinetic analyzer (SurPASS 3, Anton Paar, Austria) based on the streaming potential method. Membrane samples were cut into rectangular pieces with dimensions of 2 cm × 1 cm, and two pieces were mounted in the adjustable-gap

cell to form a parallel-plate flow channel. A KCl aqueous solution with a concentration of 0.01 mmol·L^{-1} was used as the background electrolyte.

The solution pH was adjusted to the desired values by the addition of small amounts of NaOH or HCl. Zeta potential values were calculated from the measured streaming potential using the instrument software. Each measurement was repeated three times, and the reported values represent the average of three independent measurements.

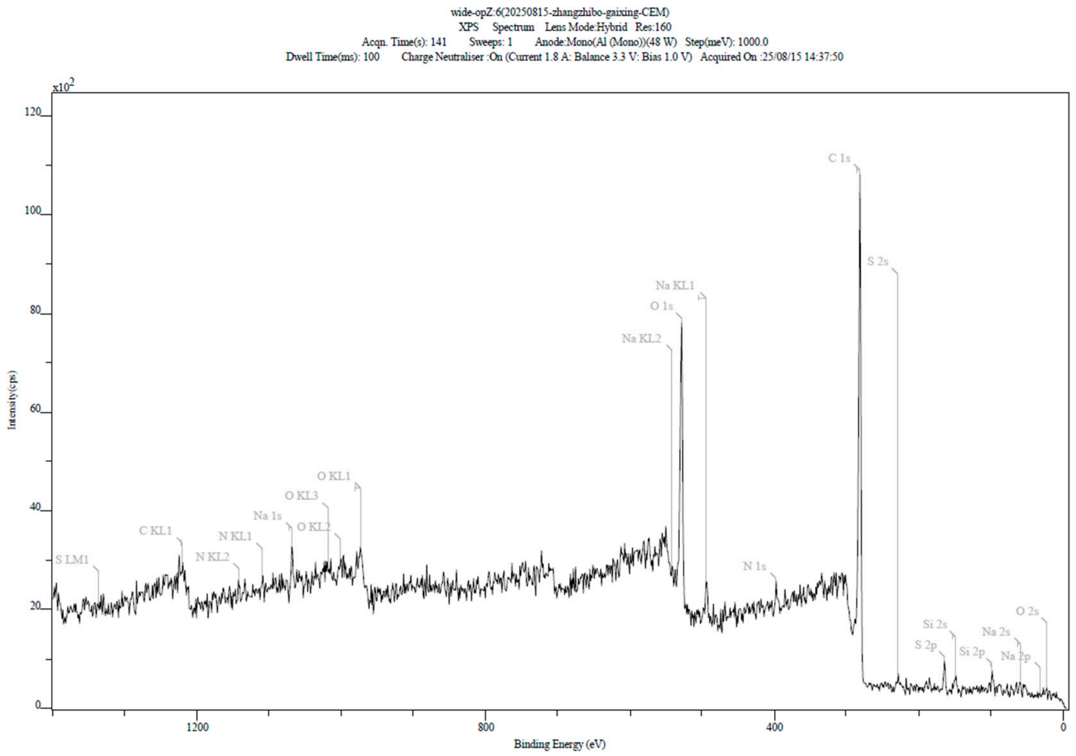

Figure S3. The general XPS survey

The chemical structure of the membrane surface was analyzed using a Fourier transform infrared spectrophotometer (ATR-FTIR, Nicolet 6700 Spectrometer,USA). Spectra of dry samples wereobtained within the range of 500-4000 cm^{-1}.

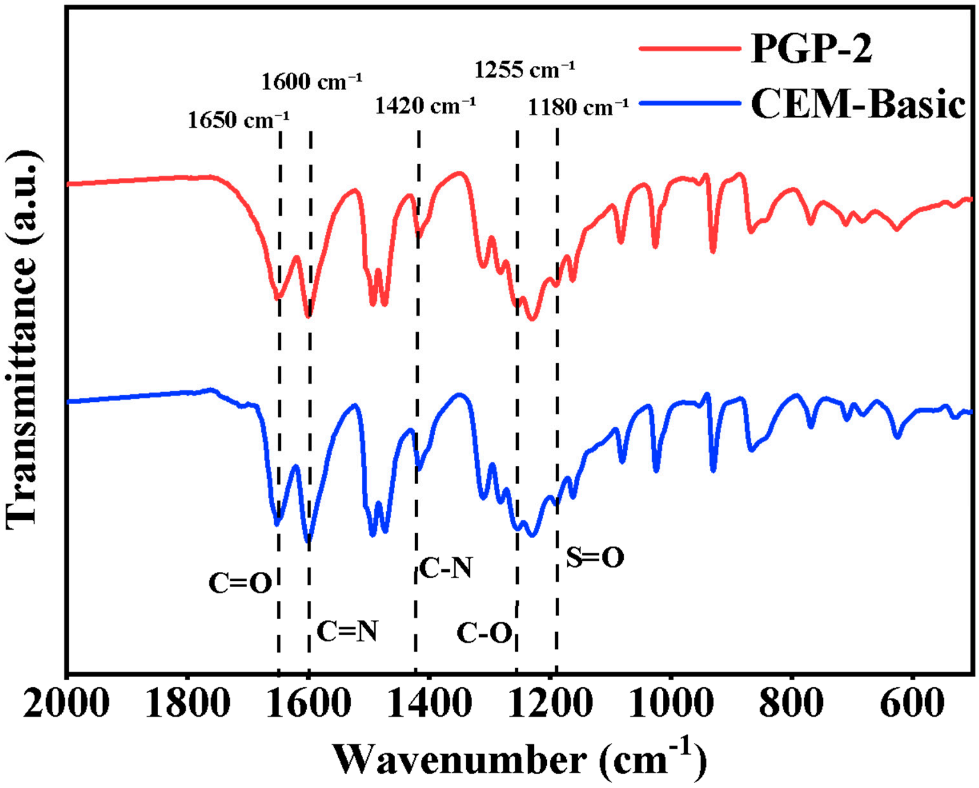

Figure S4. ATR-FTIR spectra of the prepared membranes.

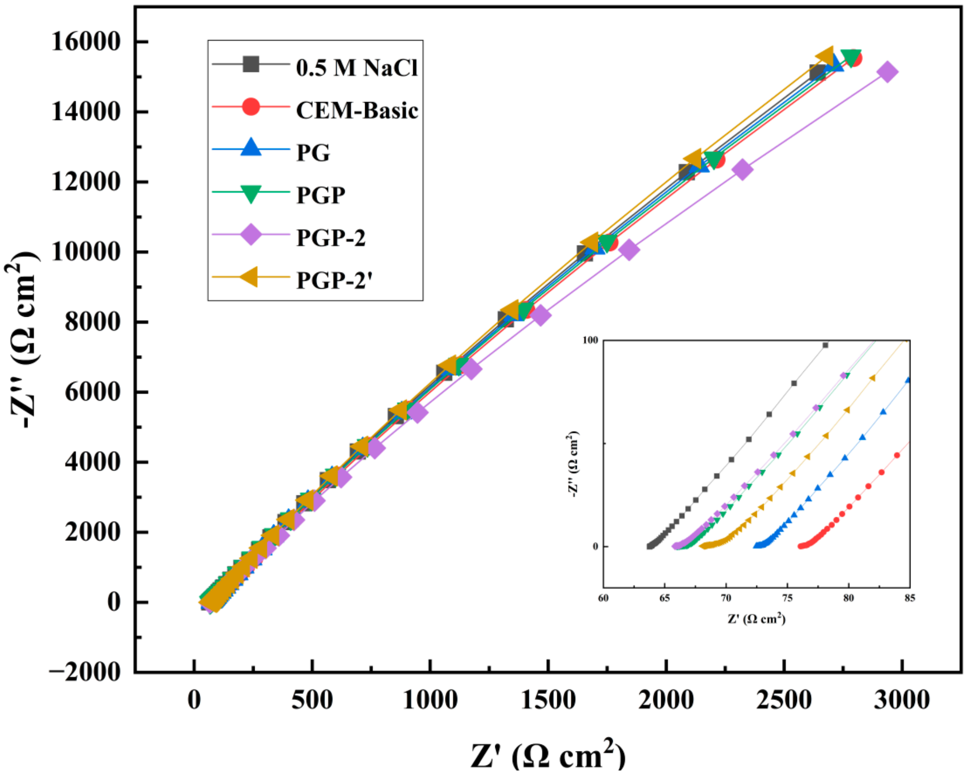

Figure S5. Representative Nyquist plot obtained from AC impedance measurements for the determination of membrane resistance.

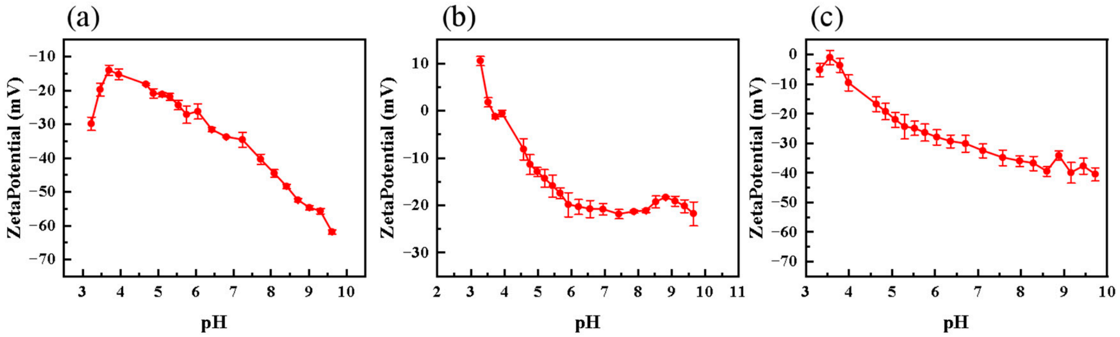

Figure S6. pH-dependent zeta-potential of pristine and modified membranes.(a) CEM-Basic,(b) PG (c)PGP-2’

Table S4. Comparison of literatures

Membrane / Modification	[Li^+]feed	[Mg^{2+}]feed	Li^+/Mg^{2+} Selectivity	Conditions	Ref.
PEI-GA-PSS sequential electro-assembly on commercial CEM	0.166 M	1.23 M	~ 108	Salt lake brine under real conditions	This work
M-Glu@PIP/PEI selective ED membrane	0.02 M	0.50 M	~ 31.83	Simulated SLB ED	[8]
ZPEI-TMC/SPEEK CEM	0.01 M	0.1 M	~ 12.43	Binary Li/Mg ED	[9]
DOPE-interlayer NF	0.01 M	0.5 M	~ 35.4	NF separation test	[10]
PIM-DB18C6-TB	/	/	~ 24.35	ED, model solutions	[11]
PAA-PA composite	0.02 M	0.4M	~ 43.3	ED tests	[12]
Positively charged NF (PEI/TMC+BTAB)	0.01 M	0.50 M	~ 95.9	Simulated brine NF	[13]
LATP-CA composite membrane	0.02 M	0.4 M	39.4 → 467	Electro-driven brine tests	[14]
Multipass NF	0.01 M	0.5 M	~1000	Brine recirculation NF	[15]
LbL hollow fiber NF	/	/	~2–3 (rejection ratios)	Modal solutions	[16]
Materials & mechanisms	various	various	~ 42	NF review trends	[17]

References

[1]S. E. Rodriguez-Cruz, R. A. Jockusch and E. R. Williams,Hydration Energies and Structures of Alkaline Earth Metal Ions, $M^{2+}(H_2O)n$, n = 5–7, M = Mg, Ca, Sr, and Ba[J],*Journal of the American Chemical Society*,**1999**,121:8898-8906.

[2]S. B. Rempe, L. R. Pratt, G. Hummer, J. D. Kress, R. L. Martin and A. Redondo,The Hydration Number of Li^+ in Liquid Water[J],*Journal of the American Chemical Society*,**2000**,122:966-967.

[3]E. R. Nightingale, Jr.,Phenomenological Theory of Ion Solvation. Effective Radii of Hydrated Ions[J],*The Journal of Physical Chemistry*,**1959**,63:1381-1387.

[4]R. Kingsbury,A guide to ion separations for the global energy transition[J],*Joule*,**2025**,9:102134.

[5]M. Sun, C. Mu, S. Wang, J. Bi, X. Guo, S. Wang and Y. Zhao,Monovalent cation exchange membranes prepared by Fe^{3+}-assisted coupled surface modification of PEI-PPy for potassium extraction from simulated brine[J],*Desalination*,**2024**,586:117835.

[6]S. Chen, C. Mao, B. Hu, W. Zhang and H. Deng,Simultaneous improvement of flux and monovalent selectivity of multilayer polyelectrolyte membranes by ion-imprinting[J],*Desalination*,**2022**,

[7]T. Mu, Z. Zhang, L. Guo, G. Xu, J. Liao, J. Weng, J. Shen and C. Gao,Quaternized poly(arylene piperidine) anion exchange membranes with enhanced alkaline stability and desalination performance[J],*Desalination*,**2025**,616:119354.

[8]W. Wang, C. Wang, R. Huang, G. Hong, Y. Zhang, X. Zhang and L. Shao,Boosting lithium/magnesium separation performance of selective electrodialysis membranes regulated by enamine reaction[J],*Water Research*,**2025**,268:122729.

[9]H. Qian, G. Xu, S. Yang, E. H. Ang, Q. Chen, C. Lin, J. Liao and J. Shen,Advancing Lithium–Magnesium Separation: Pioneering Swelling-Embedded Cation Exchange Membranes Based on Sulfonated Poly(ether ether ketone)[J],*ACS Applied Materials & Interfaces*,**2024**,16:18019-18029.

[10]Q. Zhang, W. Liu, X. Wang, W. Ding, G. Han and S. Liu,Preparation and performance study of highly permeable and selective lithium-magnesium separation nanofiltration membranes modified by phospholipid interlayer[J],*Separation and Purification Technology*,**2025**,377:134307.

[11]M. Yong, Y. Yang, L. Sun, M. Tang, Z. Wang, C. Xing, J. Hou, M. Zheng, T. F. M. Chui, Z. Li and Z. Yang,Nanofiltration Membranes for Efficient Lithium Extraction from Salt-Lake Brine: A Critical Review[J],*ACS Environmental Au*,**2025**,5:12-34.

[12]F. Wang, K. He, Z. Wang, H. Ma, F. Shi, Z. Li, X. Zhou, Q. Wu, D. Acharya, C. M. Doherty, M. R. Hill, Z. Li and H. Wang,Asymmetric design of ion-transport channels in a polymeric membrane for lithium-ion sieving[J],*Journal of Membrane Science*,**2026**,738:124807.

[13]G. Zhao, J. Sun, G. Tang, G. Pan, H. Yu, Y. Li, Y. Zhang and Y. Liu,Highly selective Mg^{2+}/Li^+ separation membranes prepared by surface grafting of a novel quaternary ammonium bromide[J],*Separation and Purification Technology*,**2024**,335:126184.

[14]D. Seo, J. Lee, S. R. Kong, G. Sim and Y. Park,Selective lithium extraction from salt-lake brine using LATP-incorporated cellulose membranes in electrically driven systems[J],*Journal of Membrane Science*,**2025**,718:123676.

[15]R. Wang, R. Alghanayem and S. Lin,Multipass Nanofiltration for Lithium Separation with High Selectivity and Recovery[J],*Environmental Science & Technology*,**2023**,57:14464-14471.

[16]D. Koukoufilippou, I. L. Liakos, G. I. Pilatos, N. Plakantonaki, A. Banis and N. K. Kanellopoulos,Separation of Magnesium and Lithium Ions Utilizing Layer-by-Layer Polyelectrolyte Modification of Polyacrylonitrile Hollow Fiber Porous Membranes[J],*Materials*,**2024**,17:

[17]S. Das, E. Deng, A. M. Tandel, S. Singh, J. V. Mowatt and H. Lin,Nanofiltration Membranes for Li^{+}/Mg^{2+} Separation: Materials and Mechanisms[J],*Journal of Polymer Science*,**2025**,64:129-162.
